# Evaluation of Stroke Risk in Patients With Atrial Fibrillation Using Morphological and Hemodynamic Characteristics

**DOI:** 10.3389/fcvm.2022.842364

**Published:** 2022-04-29

**Authors:** Lingfeng Wang, Zidun Wang, Runxin Fang, Zhi-Yong Li

**Affiliations:** ^1^School of Biological Science and Medical Engineering, Southeast University, Nanjing, China; ^2^Division of Cardiology, The First Affiliated Hospital of Nanjing Medical University, Nanjing, China; ^3^School of Mechanical, Medical and Process Engineering, Queensland University of Technology (QUT), Brisbane, QLD, Australia

**Keywords:** atrial fibrillation, left atrial appendage, stroke, hemodynamics, computational fluid dynamics

## Abstract

**Background:**

It is well known that the thrombus triggering stroke in patients with atrial fibrillation (AF) mainly comes from the left atrial appendage (LAA). This study aims to characterize the morphological and hemodynamic parameters and evaluate their differences between AF patients with and without a stroke history.

**Methods:**

Cardiac CT images were obtained from AF patients with (*n* = 10) and without a history of stroke (*n* = 10). 3D models of the left atrium (LA) were reconstructed by processing the CT image, and the LA/LAA morphological parameters were measured. Computational fluid dynamics (CFD) simulations were performed to calculate the hemodynamic parameters in LA. The species-transport model and discrete phase model (DPM) were applied to analyze blood residual ratio and particle residual ratio, two qualitative parameters for thrombus formation and flow-out potential, respectively.

**Results:**

There were significant differences in LAA actual depth (*p* = 0.002), and direct length (*p* = 0.049) between the non-stroke and stroke groups. Significant differences were also found in certain hemodynamic parameters. The blood residual ratio in LAA was significantly smaller in the stroke group than in the non-stroke group (*p* < 0.05). Moreover, the particle residual ratio within LAA was significantly smaller in the stroke groups than in the non-stroke group (*p* < 0.05).

**Conclusion:**

There are significant differences in both morphological and hemodynamic parameters between AF patients with and without a stroke history. A high blood residual ratio in LAA confirms that thrombus is more likely to form in AF patients. A significantly smaller particle residual ratio in the stroke group may suggest the thrombus formed with LAA is more likely to flow out of LAA, leading to a higher risk of stroke. The proposed morphological and hemodynamic parameters may be additional risk factors that can be used to better risk stratify AF patients.

## Introduction

Atrial fibrillation (AF) is the most common arrhythmia, and the prevalence is more than 33 million worldwide (1). Additionally, the occurrence of AF is predicted to increase rapidly as the average age of the population grows (2). Moreover, AF is well known as an independent risk factor for stroke (3, 4). It was reported that 15–18% of stroke is related to AF (5), and the autopsy study revealed that the thrombus inducing stroke in patients with AF mainly originates from the left atrial appendage (LAA) (6, 7), which is a tubular structure derived from the wall of the left atrium (LA) (8, 9).

LAA is normally categorized into four different morphologies (namely Cactus, Chicken Wing, Windsock and Cauliflower) (10–12), which are believed to be associated with different levels of stroke risk. However, it is a subjective classification with a high intro-observer difference. An objective morphological classification method is needed to quantify the association between LAA morphology and stroke risk.

Meanwhile, a non-invasive and repeatable method for simulating various fluid dynamic conditions, computational fluid dynamics (CFD) is widely applied in the cardiac hemodynamic analysis. Early studies on the CFD model of the LA and LAA were based on ideal models and subject-specific models reconstructed from CT data (13–15). A few hemodynamic parameters of patient-specific models, such as velocity, pressure, vorticity, and wall shear stress have been reported (16–18). The hemodynamic parameters in the LAA with the four different LAA morphologies have been discussed (19–21). Camara and colleagues (22, 23) investigated the change in flow field after LAA occlusion and performed a sensitivity analysis to identify the most relevant LA and LAA morphological parameters that are associated with blood flow dynamics. However, there were not intuitive descriptions of the thrombosis risk in previous studies.

To this end, this study aims to develop an objective morphological parameterization method and an intuitive description of the thrombogenesis risk. The species-transport model and discrete phase model (DPM) were applied to calculate the blood residual ratio and particle residual ratio, two qualitative parameters for thrombus formation and flow-out potential, respectively. Patients with persistent AF were divided into the non-stroke group and stroke group. Their LA/LAA morphological parameters and hemodynamic parameters were compared.

## Materials and Methods

### Study Population

Twenty patients with persistent AF from Jiangsu Province Hospital were included in this study. The patients were divided into two groups based on their stroke history: non-stroke group (*n* = 10) and stroke group (*n* = 10). The inclusion criteria were patients with persistent atrial fibrillation who underwent cardiac CT imaging before AF ablation. The exclusion criteria were: (1) valvular heart disease; (2) non cardioembolic stroke; (3) incomplete left atrium models due to insufficient cardiac CT imaging qualities. All the patients were on the blood thinner and the cardiac CT examination was taken timely for the stroke group after their stroke. The studies involving human participants were reviewed and approved by the Southeast University Human Research Ethics Committee with all participants providing written informed consent.

Images were acquired by using CT systems (Somatom Force and Definition; Siemens) with an ECG-gated dual-source single-energy protocol over the entire heart. The acquisition parameters were as follows: retrospective ECG-gated protocol; automatic tube voltage adjustment (Care kV; Siemens) from 80 to 120 kVp; automatic tube current modulation ranging between 150 and 400 mAs. Beta-blockers and nitroglycerine were used if necessary. Images was obtained following the administration of contrast medium (370 mg iodine/mL, Ultravist, Bayer Schering Pharma, Berlin, Germany) *via* a 20-G intravenous catheter, followed by 40 mL saline flush using a dual-head power injector. The injection duration was determined by scan time plus 8 s and the flow rate ranging from 4.0 to 6.0 mL/s was tailored to the body weight, heart rate (HR) and tube voltage.

### Geometrical Model and Morphological Parameters

**FIGURE 1:**
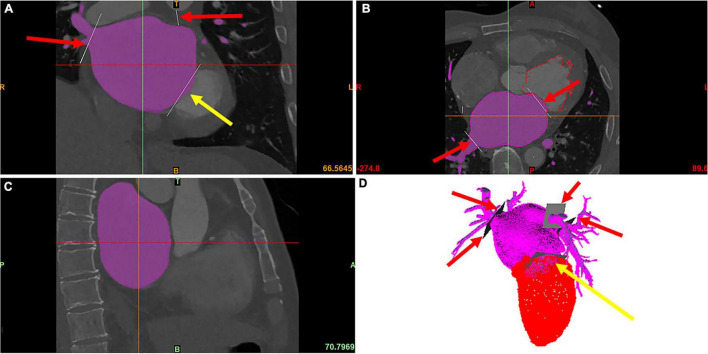
The modeling of LA and LAA. The red arrows point to PVs, and the yellow arrows point to the MV, which is cut manually. The purple area is the LA segmented using the region growing method. **(A)** The coronal image of cardiac CT. **(B)** The axial image of cardiac CT. **(C)** The sagittal image of cardiac CT. **(D)** The 3D model of LA and LV reconstructed from the cardiac CT.

The LA geometry was segmented from the cardiac CT images using Mimics Medical 21.0 (Materialise, Inc., Leuven, Belgium) (see Figure 1), and then reconstructed with Geomagic studio (Geomagic, Inc., Morrisville, the United States). The morphological parameters were measured manually using Siemens NX (Siemens Digital Industries Software, Inc., Plano, the United States).

**FIGURE 2:**
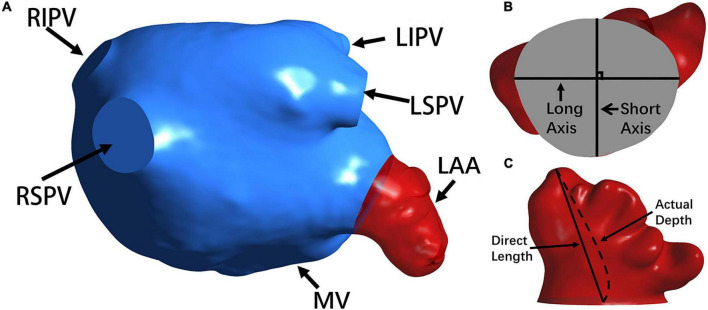
The morphology of the LA and LAA. **(A)** The LA structure. The LAA, MV and PVs (i.e., RSPV, RIPV, LSPV, and LIPV) are shown. **(B)** The long axis and short axis of the LAA orifice. **(C)** The direct length and actual depth of the LA.

In the measurement, the volume and surface area of the LA and LAA were recorded (see Figure 2A) and so were the cross section of the LAA orifice and its long and short axes (see Figure 2B). Meanwhile, to evaluate the tortuosity of the LAA, the direct length (the distance between the LAA tip and the orifice center) and the actual depth (the distance along the centerline from orifice center to the LAA tip) were measured (see Figure 2C). The tortuosity was calculated based on the LAA direct length and actual depth:


(1) $$\text{Tortuosity}=\frac{\text{ActualDepth}}{\text{DirectLength}}-1$$

### Computational Fluid Dynamics Analysis

For each patient, based on patient-specific 3D geometrical model, the pulmonary veins (PVs) were set as the velocity-inlet, and the mitral valve (MV) was the pressure-outlet. For the inlets, the velocity profile was derived from the measurement of pulmonary venous profile for a persistent-AF patient, and detailed in our former study (24) as:


(2) $$\text{V}\,\left(\text{m}/\text{s}\right)=\left\{ \begin{array}{c} 4.455\cdot \text{t},\\ -0.573\cdot \text{t}+0.553,\\ 0.9\cdot \text{t}+0.17,\\ -2.94\cdot \text{t}+1.7078,\\ 0, \end{array}\right.\;\begin{array}{c} 0\leq \text{t}\leq 0.11,\\ 0.11<\text{t}\leq 0.26,\\ 0.26<\text{t}\leq 0.4,\\ 0.4<\text{t}\leq 0.58,\\ 0.58<\text{t}\leq 0.7. \end{array}$$

where V is the flow velocity for the inlet, and t is the time in the cardiac time.

The open and close conditions of MV were set by the high- and low-pressure, which were expressed as:


(3) $$\text{P}\,\left(\mathrm{mmHg}\right)=\left\{ \begin{array}{c} 100,\\ 0, \end{array}\right.\;\begin{array}{c} 0\leq \text{t}\leq 0.26,\\ 0.26<\text{t}\leq 0.7. \end{array}$$

The diastole is from 0 to 0.26 s, and the systole is from 0.26 to 0.7 s.

Tetrahedral-element meshes were generated by Ansys Meshing using a maximum element size of 0.8 mm and a minimum size of 0.2 mm, which were confirmed to be sufficient by performing a denser mesh.

The CFD analysis were performed on the node (2*Intel Xeon E5-2680 v3) in the High-Performance Computing Center of Collaborative Innovation Centre of Advanced Microstructures using the commercial software Fluent 2019 R3 (Ansys, Inc., Canonsburg, the United States).

In the simulation, the blood was set as incompressible Newtonian fluid with density ρ = 1,060 kg/m^3^ and viscosity μ = 0.0035 Pa⋅s. The flow was simulated by the realizable k-ε turbulence model with enhanced wall treatment, and the walls were assumed as rigid and with no-slip conditions to represent the worst AF symptom. The coupled second order upwind method was used for the simulations, and the time step was set as 0.001 s.

Meanwhile, to evaluate the hemodynamics in a more intuitive way, the species-transport model was employed to study the mechanisms of thrombus formation and the DPM analysis was performed to stratify the thrombus dynamics.

For the species-transport model, the mass fraction of each species was estimated through the convective-diffusion equation:


(4) $$\frac{\partial }{\partial \text{t}}\,\left(\rho \,{\text{Y}}_{i}\right)+\nabla \cdot \left(\rho \,\vec{\text{v}}\,{\text{Y}}_{i}\right)=-\nabla {\vec{\text{J}}}_{i}$$

where *Y_i_* is the mass fraction of the ith species, and *J_i_* is the diffusive flux of the ith species, which is calculated as follows in turbulence:


(5) $$\nabla {\vec{\text{J}}}_{i}=-\left(\rho \,{\text{D}}_{i,m}+\frac{\mu_{t}}{{\text{SC}}_{t}}\right)$$

where *SC_t_* is the turbulent Schmidt number and *D*_*_i, m_*_ is the diffusion coefficient of the ith species.

For the DPM, the trajectory of discrete-phase particles is solved by integrating the particle force differential equation in the Laplace coordinate system. The force balance equation of particles in the Cartesian coordinate system is in calculated as follows:


(6) $$\frac{{\text{du}}_{p}}{\text{dt}}={\text{F}}_{D}\,\left(\text{u}-{\text{u}}_{p}\right)+\frac{{\text{g}}_{x}\,\left(\rho_{p}-\rho \right)}{\rho_{p}}+{\text{F}}_{x}$$

where *F*_*D*_(*u*−*u*_*p*_) is the drag force per unit mass of particles:


(7) $${\text{F}}_{D}=\frac{18\,\mu }{\rho_{p}\,{\text{d}}_{p}^{2}}\,\frac{{\text{C}}_{D}\,\text{Re}}{24}$$

where *u* is fluid velocity, *u_p_* is particle velocity, μ is fluid dynamic viscosity, ρ is fluid density, ρ_*p*_ is particle density, *d_p_* is particle diameter, *Re* is relative Reynolds number, and *C_D_* is drag coefficient.

For the species-transport model, the initial blood in the LA was defined as one phase, which would be replaced by the fresh blood (defined as the other phase) flowing from the PVs. The residual rate of the initial blood was calculated, and the higher blood residual ratio corresponds to the more probability of thrombus formation in the LAA. In order to study the mechanism of thrombus flowing-out from the LAA, particles were set on the LAA walls at the beginning of the third cardiac cycle, the stable flow condition. The risk of thrombus flowing-out was represented by the residual ratio of particles in the LAA, and a higher particle residual ratio corresponds to a higher ratio of particles flowing out of the LAA, and thus a higher risk of stroke.

### Statistical Analysis

Statistical analysis was performed the open-source software Jamovi. The data were tested for normal distribution using Shapiro-Wilk test and the results were described as mean ± SD or median (IQR). Independent samples *t*-test or Mann-Whitney U test was used to compare the geometrical and hemodynamic parameters between the non-stroke group and stroke group. It was considered statistically significant when *p*-value < 0.05.

## Results

### Patient Characteristics

Patient characteristics are shown in Table 1. No significant (*p* > 0.05) differences existed in age, hypertension, diabetes and left ventricular ejection fraction (LVEF) between the two groups.

**TABLE 1:** Baseline characteristic.

	Non-stroke (*n* = 10)	Stroke (*n* = 10)
Gender	9M/1F	6M/4F
Age (years)	67.9 ± 3.35	72.1 ± 2.52
CHF (*n*)	3	0
Hypertension (*n*)	7	8
Vascular disease (*n*)	2	8
Diabetes (*n*)	2	2
LVEF (%)	54.02 ± 4.42	62.43 ± 0.54

*Values are presented as mean ± SD or number of patients. CHF, congestive heart failure; LVEF, left ventricular ejection fraction.*

### Morphological Parameters

Morphological parameters of the LA and LAA are presented in Table 2. Both the actual depth and direct length of LAA were significantly larger in the non-stroke group than in the stroke group (*p* < 0.05). The surface area and tortuosity of LAA showed certain differences between the two groups but they were not statistically significant. There were no significant differences in the LA volume, LA surface area, LAA volume, LAA orifice long radius, LAA orifice short radius and LAA orifice ovality (the ratio of long radius to short radius).

**TABLE 2:** Morphological parameters.

	Non-stroke (n = 10)	Stroke (*n* = 10)	*p*-value
**LA dimensions**			
Volume (mm^3^)	138,033 (129,640–156,851)	156,811 (136,167–189,510)	0.393^†^
Surface area (mm^2^)	14,379 (13,865–15,678)	15,336 (13,505–17,169)	0.853^†^
**LAA dimensions**			
Volume (mm^3^)	11,282 (10,312–12,218)	9,903 (6,475–15,065)	0.579^†^
Surface area (mm^2^)	3,826 ± 678	3,156 ± 872	0.058*
**LAA orifice**			
Long axis (mm)	36.6 ± 3.87	34.2 ± 5.03	0.261*
Short axis (mm)	25.3 ± 2.72	24.8 ± 5.06	0.779*
Ovality	1.46 (1.37–1.52)	1.47 (1.38–1.52)	0.971^†^
**LAA tortuosity**			
Actual depth (mm)	42.8 (37.6–49.6)	31.8 (29.9–35.3)	**0.002** ^†^
Direct length (mm)	37.9 ± 10.9	29.8 ± 5.01	0.049*
Tortuosity	0.136 (0.0811–0.323)	0.0823 (0.0568–0.148)	0.190^†^

Values are presented as mean ± SD or median (IQR); p-values display significance between the non-stroke group and stroke group. *Independent samples t-test.

*^†^Mann-Whitney U-test. The bold values mean “significant differences”.*

### Blood Residual Ratio

**FIGURE 3:**
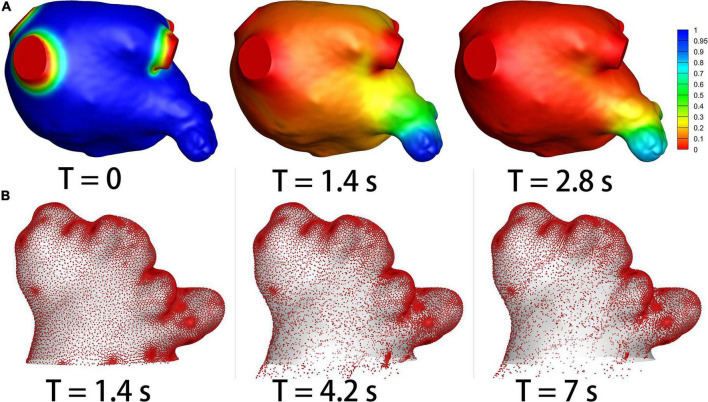
The results of species-transport and DPM models. **(A)** The contour of the initial blood. The initial blood show in blue, and the fresh blood show in red. **(B)** The particle distribution.

The residual ratios of initial blood at the end of each cardiac cycle in the LA and LAA were recorded, which represent how efficient the blood clears out from LAA at each cardia cycle. An example of the simulation results of the blood residual ratio is shown in Figure 3A. The ratios were compared between the non-stroke and stroke groups in Table 3.

**TABLE 3:** Results of species-transport model.

	In the LA			In the LAA		
	Non-stroke	Stroke	*p*-value	Non-stroke	Stroke	*p*-value
R1 (%)	12.6 (10.2–16.6)	12.7 (8.67–18.4)	0.684^†^	65.7 ± 15.6	53.5 ± 9.76	0.049*
R2 (%)	4.51 (3.09–6.53)	3.32 (2.20–4.32)	0.105^†^	42.8 ± 16.6	21.7 ± 7.64	0.002*
R3 (%)	2.55 (1.78–4.57)	1.37 (0.815–1.51)	**0.005^†^**	31.2 ± 15.6	9.26 ± 4.59	<0.001*
R4 (%)	1.80 (1.24–3.44)	0.510 (0.282–0.707)	**<0.001^†^**	20.6 (14.6–31.7)	2.91 (2.35–5.20)	**<0.001^†^**
R5 (%)	1.27 (0.915–2.17)	0.185 (0.105–0.358)	**<0.001^†^**	15.6 (10.5–24.7)	1.12 (0.785–2.43)	**<0.001^†^**
R6 (%)	0.970 (0.665–1.51)	0.0700 (0.0400–0.138)	**<0.001^†^**	12.6 (7.56–19.6)	0.435 (0.260–1.16)	**<0.001^†^**
R7 (%)	0.785 (0.480–1.21)	0.0300 (0.0100–0.0575)	**<0.001^†^**	10.4 (5.41–15.6)	0.170 (0.0825–0.575)	**<0.001^†^**
R8 (%)	0.645 (0.342–0.992)	0.0100 (0–0.0200)	**<0.001^†^**	8.59 (3.79–12.6)	0.0650 (0.0250–0.280)	**<0.001^†^**
R9 (%)	0.535 (0.230–0.830)	0.00500 (0–0.0100)	**<0.001^†^**	7.17(2.54–10.1)	0.0250 (0.0125–0.145)	**<0.001^†^**
R10 (%)	0.440 (0.0160–0.673)	0 (0–0.00750)	**<0.001^†^**	6.02(1.76–8.35)	0.0100 (0.00250–0.0700)	**<0.001^†^**
T20(s)	0.378 (0.337–0.449)	0.390 (0.312–0.495)	0.912*	3.17 ± 1.29	1.48 ± 0.266	<0.001*
T15(s)	0.452 (0.393–0.807)	0.469 (0.370–0.831)	0.853*	3.89 ± 1.46	1.73 ± 0.367	<0.001*
T10(s)	0.863 (0.591–0.930)	0.836 (0.513–0.964)	0.739*	4.99 ± 4.76	2.07 ± 0.470	<0.001*

Values are presented as mean ± SD or median (IQR); p-values display significance between the non-stroke group and stroke group. *Independent samples t-test. ^†^Mann-Whitney U-test. R1, R2, …, R10 means the residual ratios of initial blood at the end of 1st, 2nd, …, 10th cardiac cycle; T20, T15, T10 means the time when the residual ratios of initial blood reduced to 20, 15 and, 10%. The bold values mean “significant differences”.

As shown in Table 3, from the third cycle, the residual ratios for the LA were significantly smaller in the stroke group than in the non-stroke group (*p* < 0.05). While for the LAA, the residual ratios were significantly smaller in the stroke group than in the non-stroke group (*p* < 0.05) from the first cycle.

Meanwhile, we also calculated the time when the residual ratios of the initial blood reduced to 20, 15, and 10%. No significant differences existed in the time parameters in the LA (*p* > 0.05) between the two groups, but the time parameters for LAA were significantly larger in the stroke group than in the non-stroke group (Table 3).

### Particle Residual Ratio

DPM analysis was used to quantify the efficiency of thrombus particles within LAA flowing out of LAA. Figure 3B shows an example of the particle distributions within LAA in a patient case. The residual ratio of the particles in LAA at each cardiac cycle was recorded, which are shown in Table 4. The particles were released at the third cardiac cycle, so the results were recorded from the third cycle. No significant (*p* > 0.05) differences existed in the residual ratios of particles in LAA between the two groups at the beginning three cycles, but the particle residual ratios became significantly smaller in the stroke groups than in the non-stroke group (*p* < 0.05) from the fourth cardiac cycle.

**TABLE 4:** Particle residual ratio in LAA.

	Non-stroke (*n* = 10)	Stroke (*n* = 10)	*p*-value
PRR3 (%)	0.900 ± 0.0490	0.934 ± 0.0418	0.112
PRR4 (%)	0.840 ± 0.0651	0.835 ± 0.0517	0.848
PRR5 (%)	0.785 ± 0.0813	0.728 ± 0.0595	0.093
PRR6 (%)	0.734 ± 0.0914	0.646 ± 0.0655	**0.024**
PRR7 (%)	0.689 ± 0.0980	0.586 ± 0.0690	**0.014**
PRR8 (%)	0.643 ± 0.0966	0.539 ± 0.0699	**0.013**
PRR9 (%)	0.598 ± 0.0940	0.501 ± 0.0696	**0.017**
PRR10 (%)	0.547 ± 0.0879	0.466 ± 0.0654	**0.031**

*Values are presented as mean ± SD or median (IQR); p-values display significance between the non-stroke group and stroke group by independent samples t-test. PRR3, PRRM4, …, PRR10 means the residual ratios of particles at the end of the 3rd, 4th, …, 10th cardiac cycle. The bold values mean “significant differences”.*

## Discussion

AF is a common heart disease worldwide and known as an independent risk factor for stroke. The CHA2DS2-VASc score is an effective method for diagnosing whether a patient with persistent AF needs to be anticoagulated (25). However, the patients with low scores are still at risk of stroke (26). In order to further explore the mechanism of thrombus formation and stroke risk, and develop additional risk factors to better assess the risk of stroke in AF patients, we developed a quantitative strategy to characterize the morphological and hemodynamic parameters in LA/LAA. We divided the patients with persistent AF into two groups: non-stroke group and stroke group, and measured the morphological parameters of the LA and LAA and analyzed the hemodynamic characteristics.

Our results confirmed that there were some differences in the morphological parameters of LA/LAA between the non-stroke and stroke groups. Compared with the non-stroke group, the LAAs of the stroke group had a smaller actual depth and direct length. The results are consistent with a previous study by (27), who also proposed the feasibility of using morphological parameters for clinical diagnosis of AF patients. A combination of the CHA2DS2-VASc score and morphological parameters may provide a more accurate guide to diagnose whether patients with persistent AF need to be anticoagulated.

For the first time, we simulated the blood flow renewal process in the LA by applying the species-transport model. The results showed that the blood flow in the LA and LAA was more likely to be renewed in the stroke group than in the non-stroke group. This seems to contradict our conjecture that stagnant blood results in blood clots and stroke group should have a lower renewal ratio (higher blood residual ratio) based on the traditional hypothesis. One possible explanation may be that although the stroke group has a lower blood residual ratio, the residual ratios in both groups were very high within their LAA and the risk of thrombus formation were both high within the LAA in patients with AF. The lower blood residual is correlated with the shorter LAA length in the stroke group. The results suggest that the LAA is an important factor of thrombosis and the morphological parameters of the LAA are more notable in the stroke groups than in the non-stroke group. It was reported that thrombus formation is likely to happen in areas with low flow rates (28), so the areas under certain velocity may be a better way to show the risk of thrombus formation. Further extensive patient studies are required to further explore the underlying mechanism.

The process of thrombus discharged from the LAA was also studied by employing the DPM analysis. Unsurprisingly, the results showed that the thrombus had a higher propensity to expel in the stroke group than in the non-stroke group. This result showed that there is a higher likelihood of thrombus flowing out of LAA in the stroke group, which may suggest that a higher discharge of thrombus in LAA may lead to a higher risk of stroke. The proposed particle analysis may act as a useful tool to assess the risk of cardioembolic stroke in patients with AF.

Some limitations may exist in our study: First, only twenty patients with persistent AF were enrolled; Second, the wall of LA/LAA was assumed to be rigid wall. It will provide more convincing results, if a larger cohort of patients is enrolled and fluid-structure-interaction method is employed.

## Conclusion

There are significant differences in both morphological and hemodynamic parameters between patients with and without a stroke history. Particularly, the LAAs of the stroke group is shorter and less-tortuous than the non-stroke group. Both groups have a high blood residual ratio in LAA which may suggest the thrombus formation is likely to happen in all the AF patients. For the blood clots formed within LAA, the flowing out risk of the blood clots is significantly higher in the stroke group, suggesting the particle residual ratio could be used as a hemodynamic parameter to assess stroke risk in AF patients. The proposed morphological and hemodynamic parameters may be additional risk factors that can be used to better risk stratify the patients with AF.

## Data Availability Statement

The raw data supporting the conclusions of this article will be made available by the authors, without undue reservation.

## Ethics Statement

The studies involving human participants were reviewed and approved by the Southeast University Human Research Ethics Committee. Written informed consent was not required for this study, in accordance with the local legislation and institutional requirements.

## Author Contributions

LW and ZW made major contribution to the writing of the article, model construction, and data analysis. Z-YL was responsible for overall design and execution of the research. RF worked on CT image segmentation and morphological parameters measurement. All authors contributed to the article and approved the submitted version.

## Conflict of Interest

The authors declare that the research was conducted in the absence of any commercial or financial relationships that could be construed as a potential conflict of interest.

## Publisher’s Note

All claims expressed in this article are solely those of the authors and do not necessarily represent those of their affiliated organizations, or those of the publisher, the editors and the reviewers. Any product that may be evaluated in this article, or claim that may be made by its manufacturer, is not guaranteed or endorsed by the publisher.
